# Comprehensive Evaluation of RNA and DNA Viromic Methods Based on Species Richness and Abundance Analyses Using Marmot Rectal Samples

**DOI:** 10.1128/msystems.00430-22

**Published:** 2022-07-14

**Authors:** Yue Sun, Yonggang Qu, Xiaomin Yan, Guangzhi Yan, Junjie Chen, Guoshuai Wang, Zihan Zhao, Yang Liu, Changchun Tu, Biao He

**Affiliations:** a Changchun Veterinary Research Institute, Chinese Academy of Agricultural Sciences, Changchun, Jilin Province, China; b College of Animal Science and Technology, Shihezi University, Shihezi, Xinjiang Uyghur Autonomous Region, China; c Ecological Security and Protection Key Laboratory of Sichuan Province, Mianyang Normal University, Mianyang, Sichuan Province, China; d Jiangsu Co-innovation Center for Prevention and Control of Important Animal Infectious Diseases and Zoonosis, Yangzhou University, Yangzhou, Jiangsu Province, China; University of California San Diego

**Keywords:** viral metagenomics, performance comparison, species richness, species abundance, taxonomic rank, stochastic error

## Abstract

Viral metagenomics is the most powerful tool to profile viromic composition for a given sample. Different viromic methods, including amplification-free ones, have been developed, but choosing them for different purposes requires comprehensive benchmarks. Here, we assessed the performance of four routinely used methods, i.e., multiple displacement amplification (MDA), direct metagenomic sequencing (MTG), sequence-independent single-primer amplification (SIA), and metatranscriptomic sequencing (MTT), using marmot rectal samples as the templates spiked with five known viruses of different genome types. The obtained clean data were differently contaminated by host and bacterial genomes, resulting in MDA having the most, with ~72.1%, but MTT had only ~7.5% data, useful for follow-up viromic analysis. MDA showed a broader spectrum with higher efficiency to profile the DNA virome, and MTT captured almost all RNA viruses with extraordinary sensitivity; hence, they are advisable in richness-based viromic studies. MTG was weak in capturing single-stranded DNA viruses, and SIA could detect both RNA and DNA viruses but with high randomness. Due to biases to certain types of viruses, the four methods caused different alterations to species abundance compared to the initial virus composition. SIA and MDA introduced greater stochastic errors to relative abundances of species, genus, and family taxa, whereas the two amplification-free methods were more tolerant toward such errors and thus are recommendable in abundance-based analyses. In addition, genus taxon is a compromising analytic level that ensures technically supported and biologically and/or ecologically meaningful viromic conclusions.

**IMPORTANCE** Viral metagenomics can be roughly divided into species richness-based studies and species abundance-based analyses. Viromic methods with different principles have been developed, but rational selection of these techniques according to different purposes requires comprehensive understanding of their properties. By assessing the four most widely used methods using template samples, we found that multiple displacement amplification (MDA) and metatranscriptomic sequencing (MTT) are advisable for species richness-based viromic studies, as they show excellent efficiency to detect DNA and RNA viruses. Meanwhile, metagenomic sequencing (MTG) and MTT are more compatible with stochastic errors of methods introduced into relative abundance of viromic taxa and hence are rational choices in species abundance-based analyses. This study also highlights that MTG needs to tackle host genome contamination and ameliorate the capacity to detect single-stranded DNA viruses in the future, and the MTT method requires an improvement in bacterial rRNA depletion prior to library preparation.

## INTRODUCTION

Viruses are obligate intracellular parasites that nearly infect all forms of cellular life (1). As the most abundant biological entities on our planet, viruses significantly outnumber live cells with an estimated total of 10^31^ virus particles globally (2, 3). Viruses have traditionally been a concern since many of them are causative agents of diseases in humans, domestic animals, and crops. Beyond that, viruses also exert a profound influence in environmental ecology by modulating the dynamic equilibrium of microbiota with complex biochemical interaction networks (4) and in the evolution of their host by functioning as vectors of horizontal gene transfer (5). Hence, exploration of the diversity of viruses, i.e., the virome, in a variety of biospheres has tremendous merits, such as early warning of emerging infectious diseases (6), maintenance of physical health (7), understanding of the origin of life (8), and development of new biotechnological potentials (9). Since the introduction of metagenomics into the field of virology and further powered by high-throughput sequencing (HTS), viral metagenomics has greatly transformed our understanding of viral diversity and rapidly expanded our capability to profile the virome, allowing us to determine the ecological functions of the virome and virus-host interaction (1, 10, 11).

Due to the complexity of sample types and components and to the low abundance of virions, viral metagenomics usually applies pretreatments to enrich virions before viral nucleic acid (NA) extraction (12–14) and different options to increase the proportion of viral NA after extraction (12–14). Currently, several postextraction techniques are commonly used to magnify small amounts of viral NA. The linker amplification shotgun library (LASL) strategy was the first method used to amplify viral DNA in viral metagenomics, which relies on DNA fragmentation and following linker ligation before amplification (15). Sequence-independent single-primer amplification (widely termed SISPA but here we referred to as SIA) is a method related to LASL since both employ PCR to amplify viral NA prior to HTS. Instead of DNA fragmentation by shearing in LASL, SIA is originally based on endonuclease restriction of DNA followed by ligation of the adaptor linker (14). A modified version of SIA that is now widely used to screen RNA viruses (16–18) introduces a defined adaptor to viral NA by reverse transcription and double-stranded complementary DNA (dscDNA) synthesis primed by anchored random hexamers or octamers (12, 14). The multiple displacement amplification (MDA) technique employs a different principle to amplify viral DNA (19, 20), in which DNA is amplified under isothermal condition with random hexamer and high fidelity, as well as strand-displacement capacity of bacteriophage φ29 polymerase (21). Metatranscriptomics (MTT) is highly specific to the RNA virome and is widely used in RNA virus discovery, in which total RNA is extracted and followed by ribosomal RNA (rRNA) depletion prior to transcriptome sequencing (RNA-seq) (1, 22, 23). Very recently, mining viral genomes from shotgun metagenomes resulted in high-quality gut bacteriophage genomes (24, 25), indicating that direct metagenomic sequencing (MTG) of samples in viromic study without enrichment of virions and amplification of viral NA is possible. Instead of a necessary step of amplification after viral NA extraction in LASL, SIA, and MDA, total RNA and DNA are directly used for library preparation in MTT and MTG, respectively. Hence, the amplification-free MTT and MTG methods are considered the closest methods to quantitative viral metagenomic analysis, which is able to give the virus abundance based on the percentage of reads (26), although it has not been verified.

Viromic analysis, especially from an ecological view, is very sensitive to artificial bias in viral metagenomic data. Previous studies have shown that bias can be introduced into the viral metagenome in every step of sample preprocessing (27–32). Although a variety of viromic techniques have been developed, none of them can serve as a universal method to profile the complete spectrum of viruses, since these methods were designed for viruses of different genome types, which determines the predominant bias to certain virus types. Till now, several evaluations of the three amplification-based methods have been conducted, providing important insights into the advantages and disadvantages of these methods (16, 29, 33, 34). Among these DNA-specific methods, LASL is time-consuming, is preferred for double-stranded DNA (dsDNA), and requires a relatively large amount of initial input (33, 34). MDA preferentially amplifies single-stranded DNA (ssDNA), especially circular single-stranded DNA (cssDNA), and usually generates chimeric products (29, 34). Reverse transcription-based SIA has been considered to be an RNA viromic method (16, 17, 35) that is highly efficient in magnifying viral NA, but it can introduce a strong amplification bias (16, 29, 34). Furthermore, a universal disadvantage of these amplification methods is alteration of the relative abundance (RA) of viruses, although such an alteration has a minor impact on β-diversity in ecological analysis (29, 34).

Although various methods are available for viromic profiling in a given sample, choosing a rational technique for a specific purpose requires a comprehensive understanding of these methods. Previous evaluations were mainly limited to the amplification-based methods (16, 17, 29, 33). A systematic comparison of the amplification-free quasiquantitative techniques with the amplification-based methods is lacking. In addition, as capacity of HTS increasing and the cost dramatically decreasing, large-scale viral metagenomics allows us to explore the rare virosphere and is expected as an extension from descriptive analysis to ecological interpretation (36, 37), which is heavily dependent on species richness and abundance within and between samples. Thus, the robustness of these methods on rare taxa and the choice of taxonomic rank need to be considered in ecological analyses. Here, we conducted a comprehensive comparison of the popular SIA, MDA, and the amplification-free MTT and MTG using marmot rectal samples as the templates (Fig. S1); the samples were spiked with different concentrations of five quantified viruses as internal controls. The results have provided more insights into the performance of these methods and could help choose a rational technique for different virome-profiling scenarios.

10.1128/msystems.00430-22.1 FIG S1 Pipeline for the performance assessment of the four viromic techniques, which is composed of sample preparation, high-throughput sequencing (HTS), data preprocessing and virus annotation, and assessment. *, one of the three repeats of MTT, SIA, MDA, and MTG at the viral concentration of 10 gene copies/μL was used to ultradeep sequencing to assess the relationship between depth and performances. Download FIG S1, PDF file, 0.4 MB. Copyright © 2022 Sun et al. 2022 Sun et al. https://creativecommons.org/licenses/by/4.0/ This content is distributed under the terms of the Creative Commons Attribution 4.0 International license.

## RESULTS

### Host and bacterial genome contamination.

**FIG 1:**
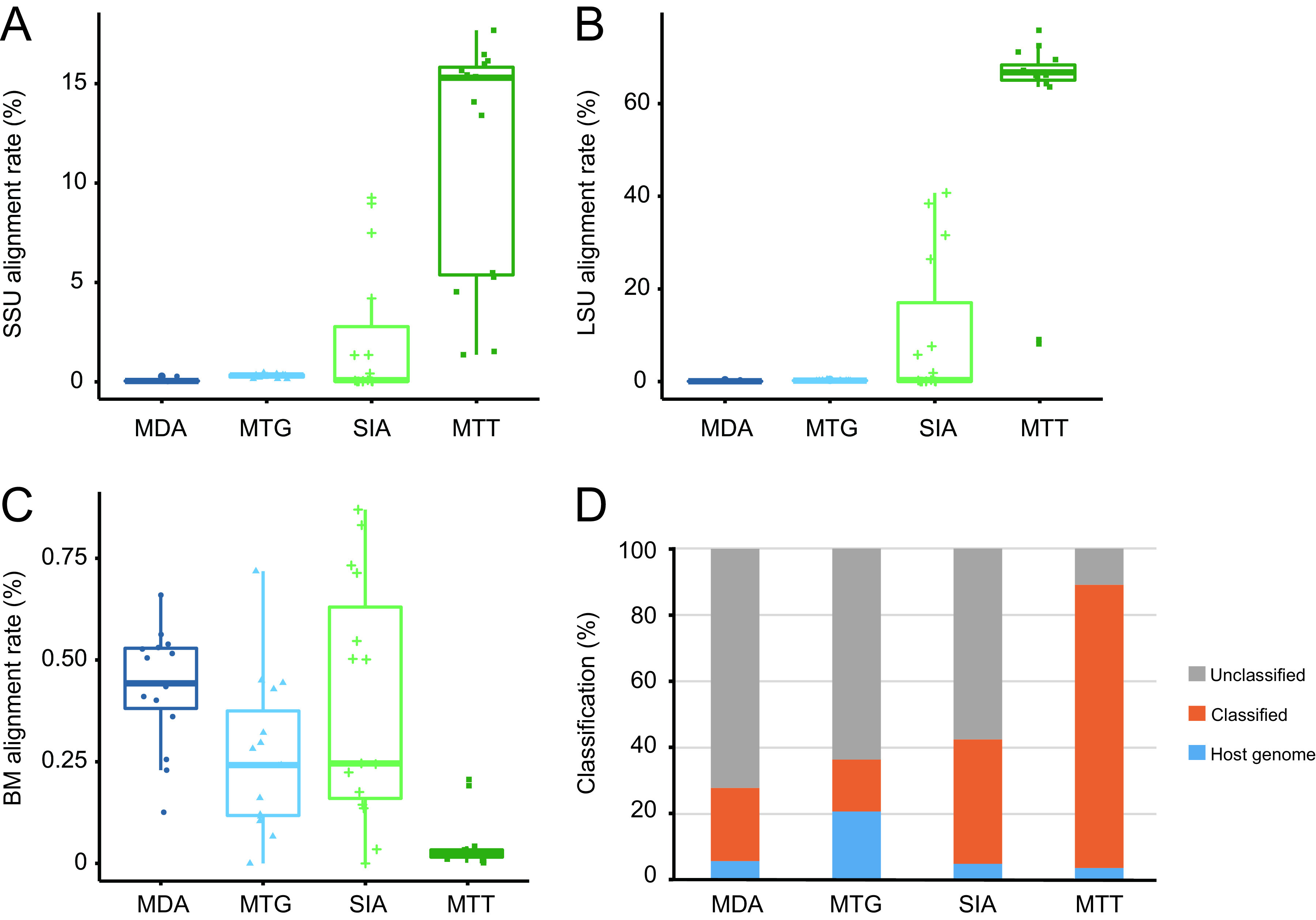
Bacterial and host contamination in high-throughput sequencing (HTS) clean data. (A to C) The alignment rates of small subunit (SSU) (A) and large subunit (LSU) (B) rRNA and bacterial marker (BM) (C) of these data were evaluated using ViromeQC 1.0. (D) These HTS data are composed of host genome, bacterial contamination (classified using Kraken2), and unclassified reads. MDA, multiple displacement amplification; MTG, direct metagenomic sequencing; SIA, sequence-independent single-primer amplification; MTT, meta-transcriptomic sequencing.

Although a variety of measures have been used in sample pretreatment to minimize contamination, host and bacterial genome contaminants are seemingly inevitable and compose a substantial part of the viromic raw data (32, 38, 39). Containing complex backgrounds of host cells and other microorganisms, as well as unknown virus composition, natural samples are ideal for determining the degree of exogenous contamination and the richness and abundance of the viromic raw data of the virus species. The template samples were generated using our archived marmot rectums and subjected to different preprocessing and library preparation according to the principles of the four techniques. All HTS data were subjected to bacterial NA contamination assessment using ViromeQC version 1.0 (40). The results showed that libraries varied dramatically in small subunit (SSU) and large subunit (LSU) rRNA alignment rates and in bacterial marker alignment rates (Kruskal-Wallis test, *P < *0.001) (Fig. 1A to C). MDA libraries showed the lowest alignment rates against SSU (~0.06%) and LSU rRNA (~0.09%) (Fig. 1A and B), and the highest bacterial marker alignment rate (0.4 ± 0.2% [mean ± standard deviation]) (Fig. 1C). SIA libraries had higher alignment rates to SSU, LSU, and bacterial markers compared to these of MTG (Fig. 1A to C). MTT libraries had the lowest alignment rates (~0.03%) against bacterial markers (Fig. 1C) but showed unexpectedly high alignment rates against SSU (12.3 ± 6.7%) and LSU (63.7 ± 25.6%) rRNA (Fig. 1A to C).

These clean data were subjected to assignment and removal of host genome and then to a taxonomic classification of bacteria, archaea, and fungi using Kraken2 (41). MTT data had the lowest assignment rates to host genome (~2.7%), followed by similar proportions in SIA (5.0 ± 4.8%) and MDA (~5.9%) libraries, but MTG libraries produced the highest ~20.8% host genomic reads (Fig. 1D). Metagenome assignment showed that MTG libraries had the lowest classification rates (15.6 ± 8.1%) and then MDA (22.1 ± 6.1%), followed by SIA (37.5 ± 30%) (Fig. 1D). Unexpectedly, MTT data had classification rates as high as 89.9 ± 30.8% (Fig. 1D), in which 98.3% were bacterial genomes (Data Set S1). After removal of these contaminated portions, MDA had the most unclassified reads (72.1 ± 17.4%) left, followed by 63.6 ± 22.7% of MTG and 57.5 ± 27.3% of SIA (Kruskal-Wallis test, *P < *0.001) (Fig. 1D). Only ~7.5% of MTT data was unassigned (Fig. 1D). These results indicate different contamination degrees of host and bacterial genomes in viromic HTS raw data with especially high proportions of host genome in MTG data and bacterial genome in MTT data.

10.1128/msystems.00430-22.6 DATA SET S1 Overview of clean data and contamination assessment. Download Data Set S1, XLSX file, 0.02 MB. Copyright © 2022 Sun et al. 2022 Sun et al. https://creativecommons.org/licenses/by/4.0/ This content is distributed under the terms of the Creative Commons Attribution 4.0 International license.

### Batch effects of MTG and MTT.

We initially failed to prepare the libraries of three replicates of MTG and MTT methods at viral concentrations of 100 and 10^4^ copies/μL, so we resampled the rectums and repeated the preprocessing, which allowed us to evaluate the batch effect of MTG and MTT. We clustered all libraries based on the MASH K-mer distances of clean and unassigned data. These libraries were well differentiated into different clusters corresponding to the methods (Fig. S2), but the rebuilt libraries of MTG and MTT detached from the initial ones, indicating that there existed a batch effect among MTG and MTT libraries. In addition, we found that one MDA library at 10^4^ copies/μL deviated from the remaining MDA ones and clustered together with the rebuilt MTG libraries, which might be due to an artificial error during library preparation. The batch effect was further examined at the virus richness level. The Jaccard distances of these libraries were calculated based on the binary virus operational taxonomic unit (vOTU) table (see next section); the results showed that the heterogeneities among MTG and MTT data were largely mitigated, but the MDA outlier still stayed away from the remaining MDA libraries. Hence, while kept in richness assessment, the rebuilt MTG and MTT libraries were excluded in virus abundance analysis in order to eliminate the heterogeneity of data imposed by the batch effect; the MDA outlier was removed from both analyses.

10.1128/msystems.00430-22.2 FIG S2 Libraries are clustered into different groups based on the MASH distances of clean and unassigned data. The rebuilt metatranscriptomic sequencing (MTT) and metagenomic sequencing (MTG) libraries are shaded. Download FIG S2, PDF file, 0.5 MB. Copyright © 2022 Sun et al. 2022 Sun et al. https://creativecommons.org/licenses/by/4.0/ This content is distributed under the terms of the Creative Commons Attribution 4.0 International license.

### Preferences of the four techniques to capture viromes of different genome types.

**FIG 2:**
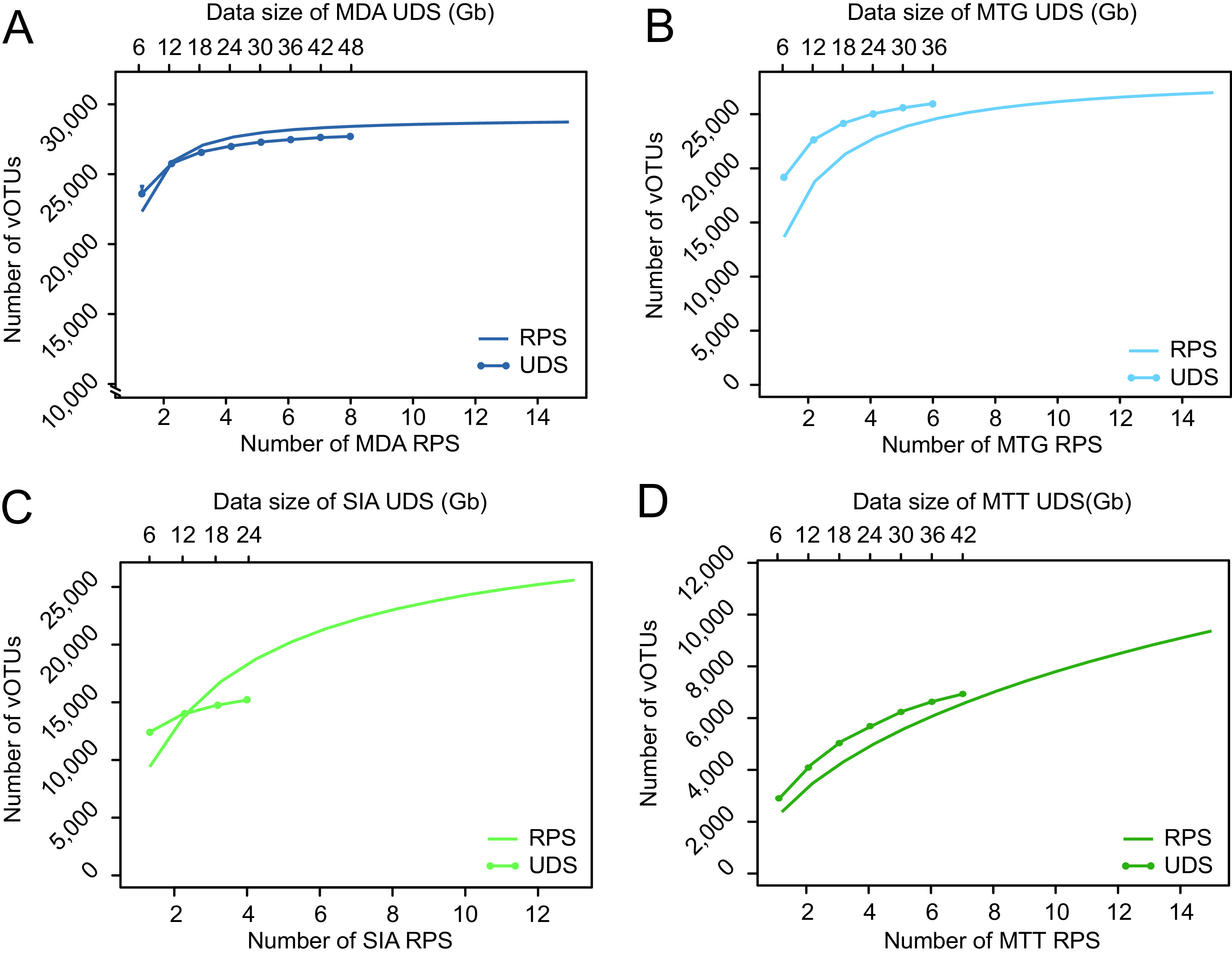
Virus operational taxonomic unit (vOTU) accumulation curves of MDA (A), MTG (B), SIA (C), and MTT (D) viromic techniques in repeated sequencing (RPS) (referring to the bottom axes) and ultradeep sequencing (UDS) (referring to the top axes).

We generated a complete viromic assemblage of the samples by coassembly of all unassigned data, which comprised 29,726 vOTUs (Data Set S2). It covered dsDNA (*n* = 27,055), ssDNA (*n* = 84), cssDNA (*n* = 2,014), double-stranded RNA (dsRNA) (*n* = 101), single-stranded RNA (ssRNA) (*n* = 271), and reverse transcribing RNA (rtRNA) (*n* = 201) viruses (Data Set S2). MDA libraries captured the most of vOTUs (95.5%, *n* = 28,398), followed by 86.1% of MTG (*n* = 25,584) and 85.6% of SIA (*n* = 25,439), but MTT detected only 27.7% of the total vOTUs (*n* = 8,242) (Fig. S3). Species accumulation assessment showed that a single library of MDA, MTG, SIA, and MTT can capture 23,432.0 ± 740.3, 13,365.9 ± 4,104.2, 9,372.0 ± 4,034.4, and 1,229.8 ± 456.0 vOTUs, respectively (Fig. 2), far from enough to profile the whole viromic assemblage. However, as sequencing repeated, vOTUs expanded significantly (Fig. 2A to D). Here, we defined whether the expansion rate of vOTUs (the proportion of the increased vOTU number to the number generated in the previous sequencing replicates) slows down to 5% and the capture rate (the proportion of the vOTU number captured in certain sequencing repeats to the total) rises to 85%; such repeats reach saturation of virome. When sequencing was repeated three, five, and seven times, the vOTU expansion rates of MDA, MTG, and SIA slowed down to 3.8, 4.3, and 4.4% and captured 95.3% (27,061.5 of 28,398), 88.3% (22,579.2 of 25,584), and 87.3% (22,216.3 of 25,439) of vOTUs (Fig. 2), respectively. However, the vOTU number captured by MTT kept increasing as sequencing repeated (Fig. 2). The results indicated that MDA is the most efficient DNA viromic technique and needs only three sequencing repeats to reach viromic saturation.

10.1128/msystems.00430-22.3 FIG S3 Virus operational taxonomic unit (vOTU) numbers of double-stranded DNA (dsDNA), single-stranded DNA (ssDNA), circular single-stranded DNA (cssDNA), reverse transcribing RNA (rtRNA), double-stranded RNA (dsRNA), and single-stranded RNA (ssRNA) captured by MDA, MTG, SIA, and MTT viromic techniques. Download FIG S3, PDF file, 0.7 MB. Copyright © 2022 Sun et al. 2022 Sun et al. https://creativecommons.org/licenses/by/4.0/ This content is distributed under the terms of the Creative Commons Attribution 4.0 International license.

10.1128/msystems.00430-22.7 DATA SET S2 Annotation of virus operational taxonomic units (vOTUs) and their read numbers in each library. Download Data Set S2, XLSX file, 7.7 MB. Copyright © 2022 Sun et al. 2022 Sun et al. https://creativecommons.org/licenses/by/4.0/ This content is distributed under the terms of the Creative Commons Attribution 4.0 International license.

The four techniques showed various preferences in viromic profiling for different viral genome types at the species richness level. MDA showed the previously recognized bias to cssDNA viruses with a capture rate of 99.2%, much higher than the next SIA of 86.5% (Fig. S3). Due to a predenaturation introduced before amplification, the modified MDA method was also permissible to detect dsDNA viruses with the highest capture rate of 96.7%. As to the linear ssDNA viruses, SIA missed only one vOTU with a capture rate of 98.8%, followed by the comparable MDA with a capture rate of 95.2% (Fig. S3). MTG captured the most rtRNA vOTUs (98.5%), far ahead of the following MDA (71.1%), which should be ascribed to the fact that these rtRNA vOTUs were predominated by the endogenous retroviral elements that were not removed by nuclease digestion prior to library preparation and preferentially recovered by MTG. Both SIA and MTT were excellent to detect RNA viruses with similar capture rates of ~86%, but each of them missed minor parts of dsRNA and ssRNA viruses (Fig. S3). Sequence analysis showed that these missed RNA vOTUs had close annotation but corresponded to different regions of target sequences.

**FIG 3:**
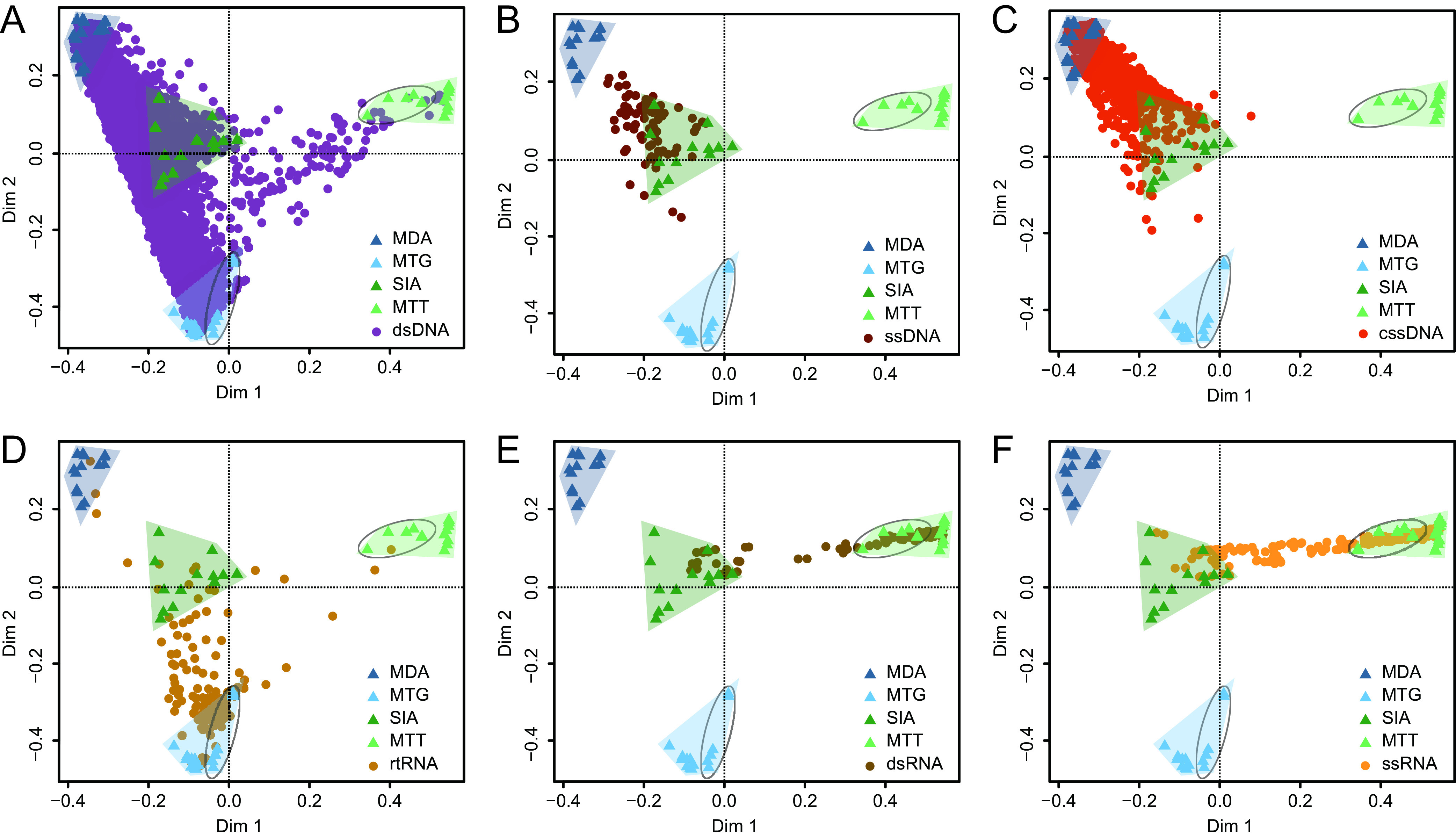
Principal coordinates analysis (PCoA) analyses revealed that libraries (triangles) aggregated into four clusters corresponding to MDA, MTG, SIA, and MTT viromic techniques that show different preferences to vOTUs (filled circles) of double-stranded DNA (dsDNA) (A), single-stranded DNA (ssDNA) (B), circular single-stranded DNA (cssDNA) (C), reverse transcribing RNA (rtRNA) (D), double-stranded RNA (dsRNA) (E), single-stranded RNA (and ssRNA) (F). The circled libraries are rebuilt MTG and MTT ones that detach from the initial ones due to batch effects.

Species abundance is a basis in the analysis of virus ecology. A Bray-Curtis dissimilarity matrix was calculated and used for principal coordinates analysis (PCoA). The 58 libraries were gathered into 4 clusters specifically corresponding to different viromic principles (Fig. 3). The MDA libraries clustered together, but due to the batch effects as assessed based on the K-mer composition of these data, the MTG and MTT libraries, respectively, gathered into two subclusters, indicating that the batch effect caused abundance variation among the data (Fig. 3). Compared to aggregations of MDA, MTT, and MTG libraries, more dispersion was noted among SIA libraries, indicating higher randomness of the method in viromic profiling (Fig. 3). The projection of vOTUs onto libraries confirmed the preferences of the four techniques for different viral genome types. Generally, MDA showed excellent performance to capture DNA viruses (Fig. 3), while MTT and SIA were highly preferential for RNA viruses (Fig. 3). These SIA libraries were distributed in the middle area of the PCoA ordination, and a lot of vOTUs from different genome types fell in this area (Fig. 3), suggesting permission of SIA to profile all kinds of viruses.

### Relative abundance variation of taxa over viromic methods and taxonomic ranks.

**FIG 4:**
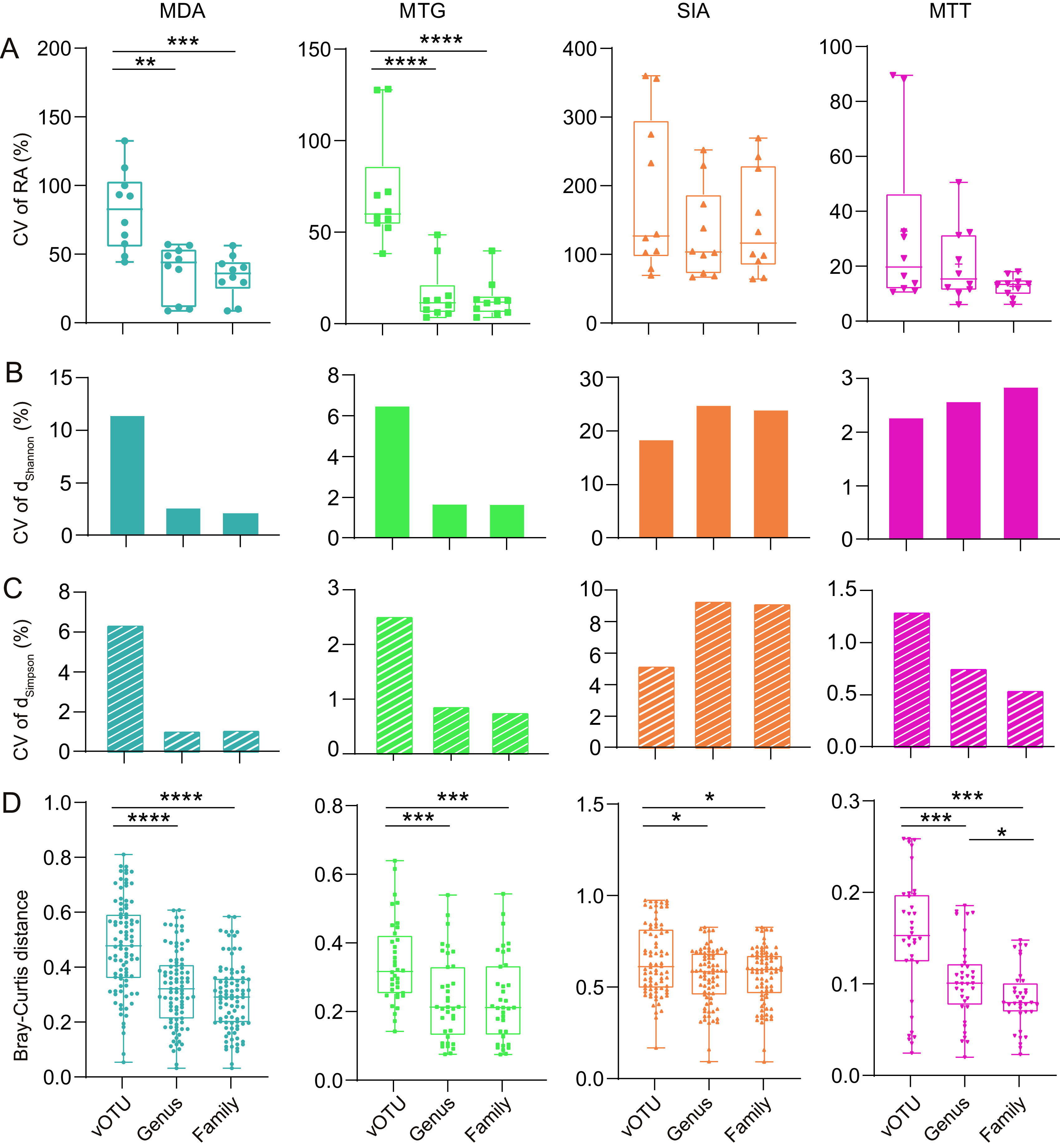
Stochastic errors of the four methods cause different variation to relative abundance (A) of taxa at the vOTU, genus, and family levels, which further affect Shannon (B) and Simpson (C) α-diversity indices and Bray-Curtis distances (D) between libraries. CV, coefficient of variation; RA, relative abundance. *, *P* < 0.05; **, *P* < 0.01; ***, *P* < 0.001; ****, *P* < 0.0001.

In viral ecological analysis, virome is often interpreted at the vOTU (species), genus, or family levels (42–44), but use of different taxonomic ranks has rarely been evaluated. In addition, dominant species are usually concerning, but the rare taxa are increasingly recognized to be more relevant to ecosystem functioning (36). Since all libraries of the four methods in this study were prepared using the same samples, the initial concentrations of viruses were the same, which was ideal to assess the impact of taxonomic rank on ecological conclusion, and the ability of these methods to capture rare taxa. These vOTUs were clustered at the approximate genus and family levels with the majority being singletons and excluded. We further removed the family taxa that shared no vOTUs with genus taxa and finally classified 1,494 and 2,250 vOTUs into 694 genus and 665 family taxa, respectively. The top 10 abundant taxa at the vOTU (those composing the genus and family taxa), genus, and family levels were extracted to calculate their RAs of reads. By comparison of the coefficient of variation (CV) of their RAs, we found that at the three levels, the RAs of SIA libraries fluctuated greatly (CV ≥ 130.7%), followed by MDA (CV ≥ 41.0%), whereas the two amplification-free methods were the most stable, with CV ≤ 13.4% at the genus and family levels (Fig. 4A; Fig. S4). With the elevation of taxonomic rank from vOTU to family, members within the taxon increased, which significantly improved the stability of RA at the genus and family levels of MDA and MTG methods. Such improvement was also noted in MTT, but it was statistically insignificant (Mann-Whitney test, *P* > 0.05). However, no improvement was noted in SIA (Fig. 4A).

10.1128/msystems.00430-22.4 FIG S4 The top 10 abundant genus taxa of each library of the four methods. A taxon name ending with “un” indicates that the taxon is unassigned by the International Committee on Taxonomy of Viruses (ICTV). Download FIG S4, PDF file, 0.5 MB. Copyright © 2022 Sun et al. 2022 Sun et al. https://creativecommons.org/licenses/by/4.0/ This content is distributed under the terms of the Creative Commons Attribution 4.0 International license.

**FIG 5:**
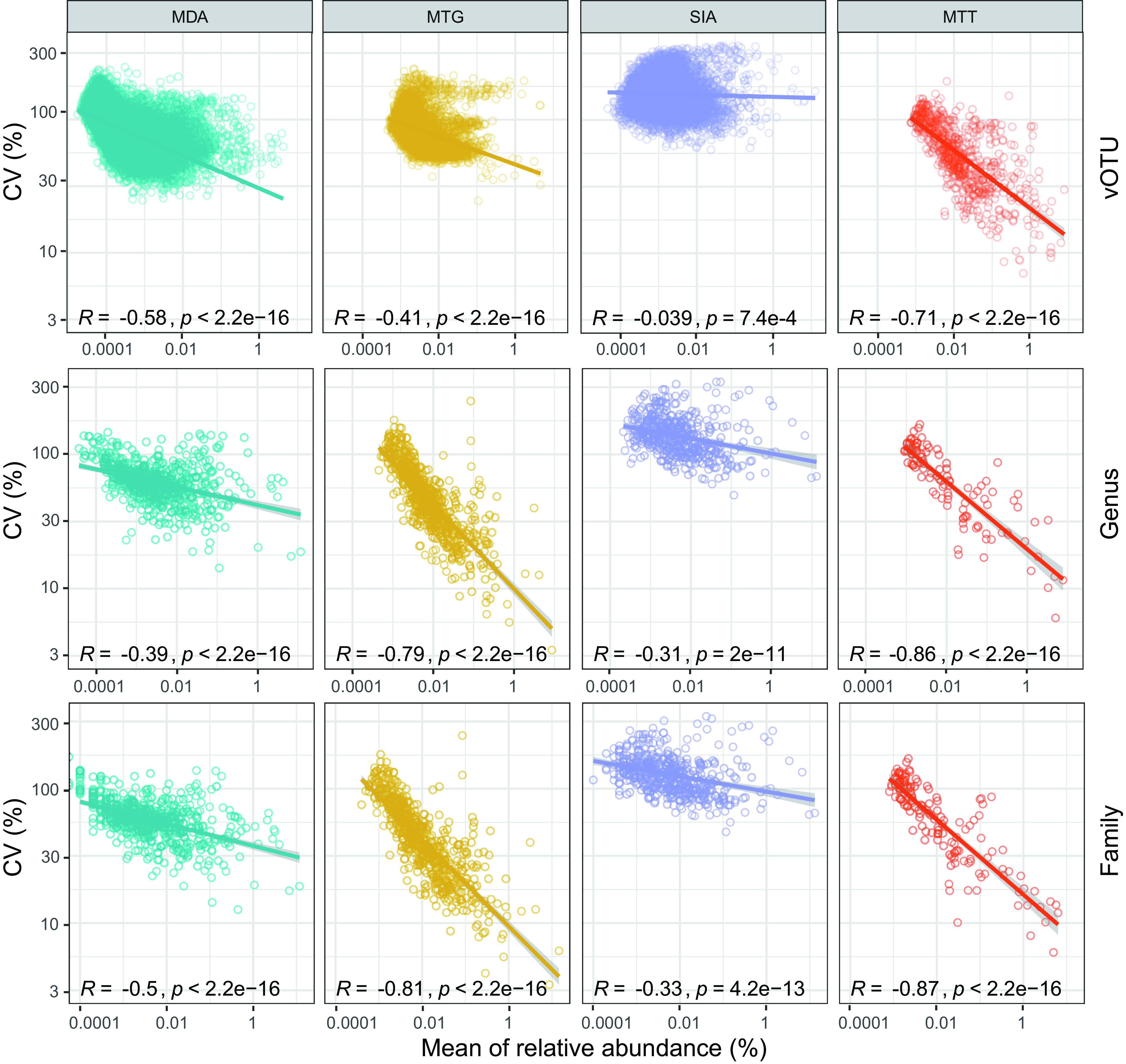
The variations in the relative abundance of vOTU, genus, and family taxa are negatively correlated with relative abundance.

We further examined whether the variation of RA at the three levels had any impact on α- and β-diversity. The Shannon and Simpson indices of the four methods at each level were calculated. The two indices of MDA and MTG were highly steady (CV < 3%) since the genus level, but that of SIA became more variable at the genus and family levels than at the vOTU level (Fig. 4B and C). The two indices of MTT were very stable (CV < 3%) at whichever level (Fig. 4B and C). The intramethod Bray-Curtis distance matrix at the three levels was compared. The viromic composition profiled by the four methods became more similar between repeats at the genus level than at the vOTU level (Mann-Whitney test, *P* < 0.05). Unlike MTT, the similarities of the MDA, MTG, and SIA methods were not further improved at the family level (Mann-Whitney test, *P* > 0.05) (Fig. 4D). These results indicated that the two amplification-free methods cause the least variation to RAs of viromic taxa compared to the amplification-based techniques. Such variation can be alleviated to different degrees at the genus level, which could ensure robust ecological conclusions as much as possible. We further profiled RA variations over taxa of the four methods at the three ranks. All methods showed similar variation patterns, i.e., variation of RA was negatively correlated with RA (*R* < −0.039, *P < *0.001) (Fig. 5). Especially the abundant taxa (RA > 1%) of MTG and MTT at the genus and family levels and those of MTT at the vOTU level had quite stable RAs with CVs close to 10%, but the RAs of those rare taxa (RA < 0.01%) of all methods across ranks were very variable, with a majority of CVs > 30% (Fig. 5).

### Bias assessment of the four methods using spiking viruses.

**FIG 6:**
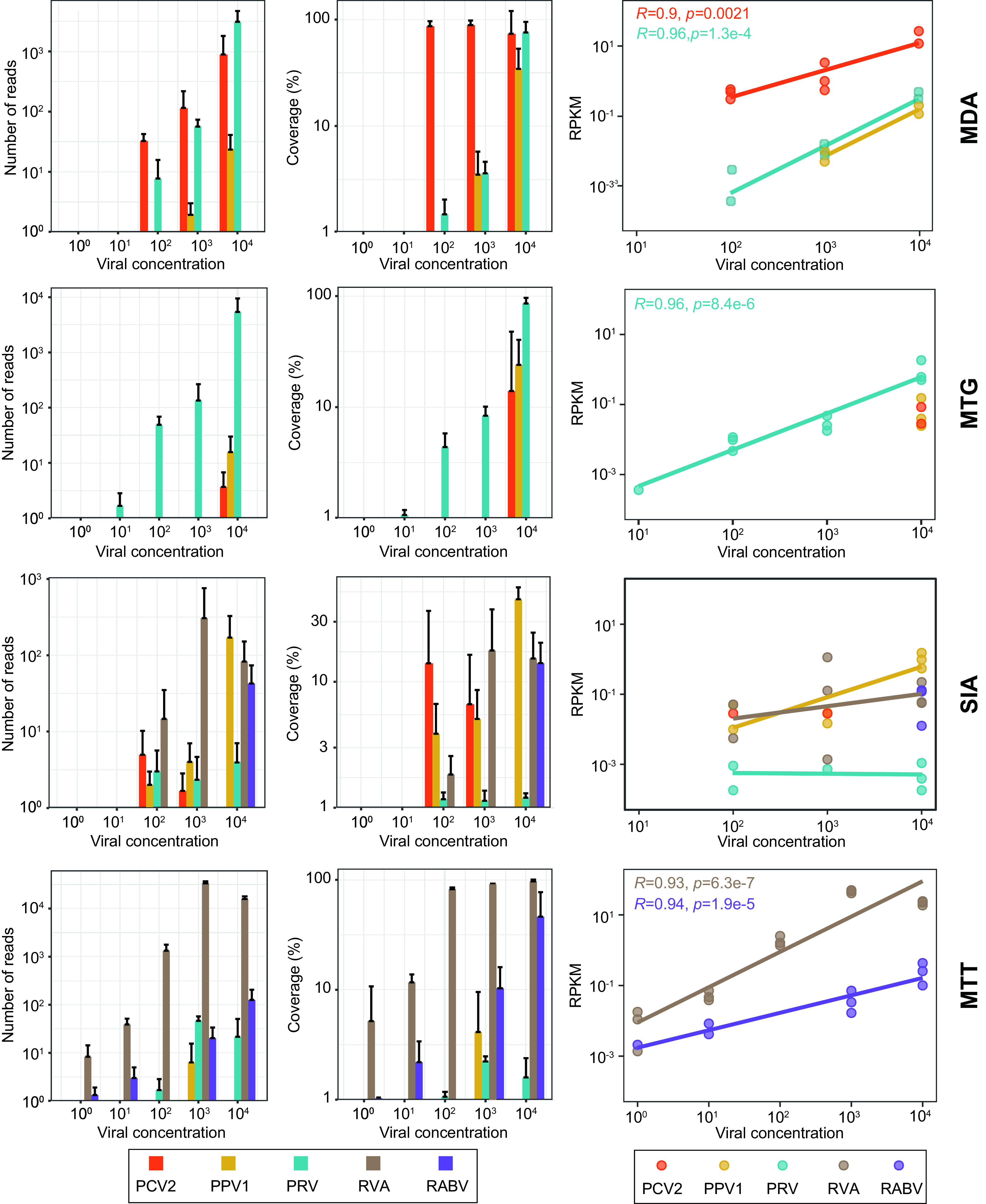
Read numbers (left column), the complete genome coverage (middle column), and the linear relationship (right column) between the read abundance and the viral concentration (gene copies/μL) of five spiking viruses in the four viromic techniques. The colors in each panel represent the different viruses, as shown in the key at the bottom of the figure. RPKM, reads per kilobase per million mapped reads; PCV2, porcine circovirus 2; PPV1, porcine parvovirus 1; PRV, porcine pseudorabies virus; RVA, group A rotavirus; RABV, rabies virus.

We used porcine pseudorabies virus (PRV), porcine parvovirus 1 (PPV1), porcine circovirus 2 (PCV2), group A rotavirus (RVA), and rabies virus (RABV) (Table 1), representing genome types of dsDNA, ssDNA, cssDNA, dsRNA, and ssRNA, respectively, as spiking viruses. These viruses were quantified and equally mixed to a series of concentrations of 1 to 104 genomic copies/μL with 10-fold dilution and then were added to marmot rectal template samples (Fig. S1). These spiking viruses in a sample had the same initial concentrations, but their ratios of read abundance (reads per kilobase per million mapped reads [RPKM]) have changed greatly. The RPKM ratios of PRV, PPV1, and PCV2 in MDA data of samples with initial concentrations of 10^3^ and 10^4^ gene copies/μL changed to 2:1:320 and 6:1:123, respectively. However, such a ratio in MTG at 10^4^ gene copies/μL changed to 100:7:4, showing a skew of MTG to dsDNA viruses. The ratio of RVA and RABV in MTT at 10^3^ and 10^4^ gene copies/μL, respectively, changed to 1,150:1 and 109:4, suggesting a skew of MTT to dsRNA viruses. Read numbers of a virus between samples significantly reflect the viral load alteration in some viromic tests. The viral RPKMs of PCV2 and PRV in MDA, PRV in MTG, and RVA and RABV in MTT kept highly linear positive change as the increase of the initial viral concentration, with a growth rate of 1.9 ± 2.0 (*R *≥ 0.90, *P ≤ *0.0021) (Fig. 6). However, such a relationship in SIA was not established statistically. This result keeps in line with the Bray-Curtis distance matrices of the four methods, in which the read abundance in SIA libraries has been greatly altered, resulting in high heterogeneity between SIA libraries (Fig. 4D).

**TABLE 1:** Details of spiking viruses used in this study^*a*^

Virus	Family	Genome type	Genome size (kb)	Accession no.
Porcine pseudorabies virus	Herpesviridae	Linear dsDNA	137,764	JF797217
Porcine parvovirus	Parvoviridae	Linear ssDNA	5,075	NC_001718
Porcine circovirus 2	Circoviridae	Circular ssDNA	1,768	KT719404
Group A rotavirus	Reoviridae	Segmented dsRNA	18,135	KC960619 to KC960629
Rabies virus	Rhabdoviridae	Linear ssRNA	11,928	AF499686

a dsDNA, double-stranded DNA; ssDNA, single-stranded DNA; dsRNA, double-stranded RNA; ssRNA, single-stranded RNA.

### Sensitivity comparison.

These serially diluted spiking viruses can serve as internal controls to assess the sensitivity of methods to detect rare species and the relationship between virus concentration and read numbers. As shown in Fig. 6, MDA and MTG could capture the three spiking DNA viruses with the former able to detect PRV and PCV2 at concentrations as low as 10^2^ copies/μL and also more efficient to obtain the complete genome, whereas the latter is more sensitive in the detection of PRV (at 10 copies/μL) but blunter in the two small ssDNA viruses PCV2 and PPV1 (detect as high as 10^4^ copies/μL). SIA could detect RVA and the three DNA viruses at 10^2^ gene copies/μL and RABV at 10^4^ copies/μL but showed a lower capability in the complete viral genome assembly, even with viral NA concentration as high as 10^4^ copies/μL. The highest genome coverage produced by SIA was 44.3 ± 11.0% (for PPV1) (Fig. 6). MTT showed outstanding detection sensitivity in the two RNA viruses (at 1 copies/μL) and can even produce enough reads to cover the complete genome of RVA at 10^2^ copies/μL (Fig. 6).

### Comparison of viromes profiled by ultradeep sequencing and replicated sequencing.

Ultradeep sequencing (UDS) can enhance the capacity to profile virome by producing an extremely large number of sequences using a single library, whereas repeated sequencing (RPS) can refresh the library with new sequences by multiple independent library construction and hence is an alternative to improve viromic profiling. One library of the four techniques at 10 gene copies/μL was selected for additional sequencing, which eventually generated 47.1, 36.8, 23.3, and 40.7 Gb of data for MDA, MTG, SIA, and MTT, respectively (Fig. 6). The data generated by UDS were randomly subsampled to 6 Gb and to its serial folds. As shown in Fig. 2, the number of vOTUs grew as the amount of data increased. As to MDA, the number of vOTUs accumulated to saturation at 18 Gb with the expansion rate going down to 2.2%, with the capture rate reaching 93.6% (26,580.7 of 28,398) (Fig. 2A). MTG saturated at 24 Gb with an expansion rate going down to 2.5% and a capture rate reaching 97.4% (24,925.3 of 25,584) (Fig. 2B). However, the vOTU numbers of SIA and MTT kept rising and captured only 61.2% (15,572 of 25,439) and 81.0% (6,676 of 8,243) of vOTUs at their final data sizes of 23.3 and 40.7 Gb, respectively (Fig. 2C and D); i.e., SIA and MTT did not reach saturation at such data sizes. Although both saturated with data size of 18 Gb, RPS of MDA showed a higher capture rate (94.3%) compared to that of UDS (92.7%), indicating that RPS is slightly more efficient than UDS in MDA. As to MTG, RPS saturated after five repeats with a capture rate of 88.3% and produced 30 Gb of data; hence UDS is more efficient because it saturated at 24 Gb with a capture rate of 97.4% (Fig. 2B). By comparison of the accumulation curve of SIA, RPS captured more vOTUs than UDS, since sequencing was repeated three times (Fig. 2C), which should be due to the high randomness of SIA in amplification, and multiple SIA pretreatments of the same sample can significantly improve the viromic coverage. However, UDS was more efficient to capture viruses than RPS in MTT (Fig. 2D), which indicates that sequence compositions kept highly homogeneous in libraries of the two amplification-free methods, enabling UDS to detect trace vOTUs.

## DISCUSSION

The recognized role of viruses has dramatically expanded from the traditional disease-causing agents to the current necessary component of microecosystem (7, 9, 45, 46). In HTS-based viromic studies, enriching virions by reducing exogenous contamination is a critical step. Exogenous contamination derives from nonviral microbes and hosts, which can be minimized by such means as ultracentrifugation, ultrafiltration, pore-sized filtration, nuclease digestion, and amplification of viral NA, with the former two often used to downsize environmental samples. In contrast, due to the limited initial sample size, the latter three methods are widely used in animal viromics. Virome quality check and component analyses of HTS clean data consistently indicate that the data sets generated by the four techniques vary in contamination of bacterial and host genomes, with MDA having the least exogenous contamination (Fig. 1A to D). MTG data contained the highest host genome contamination (Fig. 1D), inversely proving that nuclease digestion is necessary for viromic studies. Although a depletion of mammalian rRNA was implemented prior to library construction, these reads still dominate in MTT data (Fig. 1D), resulting in very little data left for viromic annotation, which indicates that an improvement to deplete rRNA, especially those bacterial rRNA, is needed.

Virus species richness and abundance are often measured in viral metagenomic analysis. Richness refers to the species number harbored in a given sample, while abundance indicates the individual number of a virus species. Preference of method is the predominant factor affecting species richness. It is well known that MDA is skewed toward cssDNA, but it also shows incomparable ability to profile all types of DNA viruses. Technically, MTG is not designed for ssDNA viruses, as it requires dsDNA to prepare HTS library. Although some cssDNA and ssDNA viruses appeared in the MTG viromes (Fig. S3), they had very low read counts and predominantly pointed to the MDA and SIA libraries (Fig. 3B and C). As we noted previously (47, 48), SIA uses reverse transcriptase and Klenow fragments to convert RNA to dscDNA, determining it a RNA viromic method (35). However, it is also compatible with DNA viruses (18, 35), due to the side activity, i.e., DNA-directed DNA polymerase, of reverse transcriptase to synthesize the counterpart of ssDNA (49), which makes it the only method that can simultaneously profile DNA and RNA viromes, although not so efficiently as the specific methods. MTT is the best RNA viromic method with excellent sensitivity (Fig. 6), which has already been proven and widely used in RNA viromic profiling (23, 50). Nonetheless, we observed a bias of MTT to dsRNA viruses (Fig. 6), which is possibly and partially because dsRNA doubles cDNA compared to ssRNA in library preparation.

Species abundance, often indicated using read RA, is critical to the ecological analysis of virome. However, read RAs are dramatically altered in all viromic analyses compared to the initial ratio of virus concentration (Fig. 6), which is determined by the principle of method and cannot be alleviated but instead accumulates as experiment repeating and thus is a systematic bias (29, 33). In addition, due to varying stability or reproducibility, the four methods also introduce stochastic errors to taxon RAs, of which SIA was the most variable, followed by MDA, while the two amplification-free methods were largely steady (Fig. S5). Stochastic error can be mitigated using repeated experiments and inclusion of more members within taxon. The former enables us to use the mean of RA to avoid stochastic fluctuation, while the latter refers to the taxonomic rank that sequences are clustered to. Currently, vOTU or species is the basic taxon often used in viromic analysis (51), but genus and family are also involved (42–44). Species provides a finer taxonomic resolution, allowing accurate identifications of ecotypes responding to environmental variables (52) and are apt to be masked at high-rank taxa that provide coarsely ecological resolution (52). However, the species level—especially the rare one—has limited tolerance to and hence is prone to be influenced by the stochastic error of the method. Comprising multiple members that enable to offset stochastic errors among them, the high-rank taxon is hence capable of being technically supported to draw a robust conclusion. However, members of the high-rank taxon are usually complicated, e.g., the families *Parvovirdae* and *Rhabdoviridae* containing viruses infecting plants and animals (53). Thus, if viromic analysis is based on such taxa, it would be difficult to interpret and understand its ecological meaning. Accordingly, an equilibrium between ecological meaning and technical robustness should be considered in viromic analysis.

Taxa at the species, genus, and family levels were assessed in this study to determine their tolerance to stochastic errors of methods. Stochastic errors caused the highest variation of RA at the species level, which was significantly mitigated at the genus level (Fig. 4A), but it was not further ameliorated at the family level (Fig. 4A), which should be ascribed to the fact that the high diversity of viruses in this study did not provide substantial member addition to family taxa compared to genus taxa, and the predominant vOTUs were rare with high variation in RA (Fig. 5) and hence contributed little to the stability improvement of family taxa. Responses of taxa at the three levels to stochastic errors further influenced α- and β-diversity (Fig. 4B to D). Generally, richness and abundance of genus taxa kept significantly higher homogeneity than that of species taxa, but such homogeneity was not further improved in family taxa (except for beta diversity among MTT libraries) (Fig. 4B to D). These results indicate that taxa at the genus level show good tolerance to stochastic errors; on this basis, the analysis can be technically supported to draw conclusions close to real situations. Moreover, a genus is a group of species sharing closer common characteristics than those in a family, such as genomic structure and size, antigenicity, and host spectrum (53); hence, a classification at the genus level meets the requirement for a conclusion with biological and/or ecological meaning. Currently, genus-level taxonomic assignment of bacteriophage genomes has been well enabled using vConTACT2, an application of gene-sharing networks (54), but classification of eukaryotic virus genomes is very complicated, since they have more diverse genomic configurations (54, 55). In addition, previous evaluation found that amplification-based viromic methods introduce stochastic errors to low-input samples (29, 56), but we found that such errors also influence rare species in high-input samples (Fig. 5). Rare species compose the rare biosphere in the microbial community and have gained increasing attention, since they contain key taxa immediately responding to environmental variables and functioning regulatory and metabolic activities in microecosystem (36). The susceptibility of rare species to stochastic errors indicates that abundance-based ecological studies should be interpreted with caution, but it has a minor impact on richness-based analyses.

Here, we also determined how UDS and RPS benefit viromic profiling of the four methods. Because of high efficiency, viromes profiled by both UDS and RPS of MDA rapidly saturated at the data size of 18 Gb (Fig. 2A). It is of note that the data size was speculated using rectal samples and can be referenced only in viromic studies of similar sample types using the same MDA protocol as this study. Since different sample types (e.g., swabs, sera, and environmental samples) have various virus concentrations and backgrounds, the data size of saturation may be different and should be independently determined. The MTT virome did not saturate with a data size of 40.7 Gb of UDS and 15 RPS replicates (Fig. 2D), which undoubtedly should be ascribed to the predominantly bacterial rRNA contamination that results in only ~7.5% of MTT data (i.e., ~450 Mb per library) that were left for RNA viromic profiling. Considering that the number of vOTUs captured by UDS exceeded that by RPS at any data size (Fig. 2D), UDS is recommended in MTT RNA viromic profiling. Furthermore, we noted that with the increase of data size, the curves of UDS and RPS converge gradually in the MTT and MTG methods (Fig. 2B and D) and are very close to each other in MDA (Fig. 2A), but there seems to be a trend to widen the superiority of RPS over UDS in SIA (Fig. 2C). This is likely due to the high randomness of SIA, which enables RPS to include many more new sequences by repeated sample processing and sequencing. Generally, UDS can be an optimal option to improve the viromic profiling in the MDA, MTG, and MTT methods; plus RPS requires repetition of sample preprocessing and library construction, which is far more costly than UDS.

In conclusion, this study provides detailed insights into the performances of the four methods, allowing researchers to choose the appropriate method for different purposes. MDA and MTT show higher efficiency and sensitivity, as well as broader coverage in DNA and RNA viromic profiling, and thus can be preferentially employed in richness-based viromic studies. Due to higher tolerance to stochastic errors, MTG and MTT are the recommended methods for abundance-based analyses, in which use of genus taxa can ensure technically supported and biologically and/or ecologically meaningful conclusions. This study also highlights necessary improvements to MTG and MTT methods in the future: the former needs to tackle host genome contamination and ameliorate the capacity to profile cssDNA and ssDNA viruses, while the latter requires an efficient measure to eliminate bacterial rRNA prior to library preparation.

## MATERIALS AND METHODS

### Virus information and quantitative real-time PCR assays.

Five viruses representing different genome types were cultured in our laboratory: RVA MSLH14, containing 11 segments of dsRNA, was cultured in rhesus monkey kidney Marc145 cells with serum-free Dulbecco’s modified Eagle’s medium (DMEM; Corning, Manassas, VA) after trypsin treatments (57). A virulent RABV strain SRV9 was cultured in baby hamster kidney-21 (BHK-21) cells supplemented with 5% fetal bovine serum (FBS; Gibco, Grand Island, NE) in DMEM. PRV, PPV1, and PCV2 were all cultured in porcine kidney-15 (PK15) cells with 8% FBS in minimum essential medium (MEM, Corning). All virus cultures were maintained in a cell incubator with 5% CO_2_ at 37°C. Previously described PCV2 and RABV TaqMan reverse transcription (RT)-PCR/PCR assays were modified or used to detect the two viruses. Primer pairs and probes of PRV, PPV, and RVA were determined using Premier 5.0. DNA or RNA of 200 μL virus culture was extracted using RaPure Viral RNA/DNA kit (MAGEN, Guangzhou, China). Quantitative RT-PCR (qRT-PCR)/quantitative PCR (qPCR) was performed using TaqMan qPCR Mix (Probe qPCR) (Monad, Suzhou, China) in a 20-μL reaction, including 10 μL qPCR mix, 10 pmol each primer, 5 pmol probe, and 1 μL template. The following PCR program was used: 40 cycles of 95°C for 15 s and 60°C for 30 s. To generate a standard curve for these assays, plasmids containing the region of interest were serially 10-fold diluted from 10e+7 to 1 copies/μL. The samples were processed in a Stratagene Mx3000P PCR machine (Agilent Technologies, Waldbronn, Germany) with distilled water as a negative control.

### Template sample preparation.

Our archived rectums of 31 long-tailed marmots (Marmota caudata) were used to generate template samples, all of which were validated to be negative for the spiking viruses using above qRT-PCR/qPCR methods. In order to start with the same template, ~0.1 g rectum with contents of each animal were cut and combined, prior to homogenization using 3 mL sterile phosphate-buffered saline (PBS) solutions spiked with five viruses at a series of concentrations (Fig. S1). After homogenization, the samples were sequentially subjected to centrifugation at 10,000 × *g* at 4°C for 5 min and filtration through 0.45-μm-pore-size membranes (Millipore, Boston, MA). The filtrates were immediately subjected to the following pretreatments. The preparation of template samples at each dilution of viral spikes was triplicated in parallel.

### Sample pretreatment and HTS.

For the SIA method, our previously published protocol was adapted in this study (47, 48). Briefly, 260 μL filtrate was subjected to free NA digestion with a mixture of DNase I and RNase A (TaKaRa, Dalian, China) and viral NA extraction using an RNeasy minikit (Qiagen, Dusseldorf, Germany). Viral NA was converted into cDNA followed by dscDNA synthesis using barcoded octamers and then randomly amplified using the defined barcode as primer. The PCR products were purified with 1 μg used to Illumina pair-end (150 bp) sequencing at an Illumina NovaSeq sequencer. For the MDA method, after digestion of free NA, viral DNA of 260 μL filtrate was extracted using a DNeasy blood and tissue kit (Qiagen) and isothermally amplified using an Illustra GenomiPhi V2 DNA amplification kit (GE, Fairfield, CT) as per the manufacturer’s manual. Viral DNA was heated to 95°C with sample buffer for 3 min and then immediately cooled on ice to denature dsDNA. After adding enzyme mix, the sample was incubated at 30°C for 1.5 h and followed by inactivation at 65°C for 10 min. The purified products were subjected to HTS in a manner similar to that described above. For MTT, 260 μL filtrate was directly subjected to total RNA extraction using TRIzol reagent (Invitrogen, Carlsbad, CA) without a prior digestion of free NA. Following rRNA depletion using the RiBo-Zero Magnetic Gold kit (Epicentra Biotechnologies, Madison, WI), the remaining RNA was subjected to RNA-seq using a NEBNext Ultra directional RNA library prep kit (NEB, Ipswich, MA). For MTG, without a prior digestion of free NA, the total DNA of 260 μL filtrate was directly extracted using the phenol and chloroform method and subjected to library preparation using NEBNext Ultra DNA library prep kit for Illumina (NEB) for HTS. About 6 Gb raw data of each library were generated. Due to the low NA concentration of MTG and MTT methods at viral concentrations of 100 and 10^4^ copies/μL, the HTS library preparation of their three replicates failed, so we resampled the rectums and repeated the preprocessing.

### Data preprocessing and viral metagenomic analysis.

All raw reads were quality checked using FastQC version 0.11.7 and trimmed using Trimmomatic version 0.38, and the resultants were used for bioinformatics analyses as clean data. The bacterial, archaeal, and fungal contaminations were assessed using ViromeQC version 1.0 (40). Host genomes were removed from clean data using Bowtie2 version 2.4.1 with the very sensitive end-to-end mode by mapping against the shotgun sequencing assembly of Marmota himalayana (GenBank accession no. GCA_005280165.1), a close relative to M. caudata (58). The remaining reads were subjected to a fast taxonomic classification of bacteria, archaea, and fungi using Kraken2 version 2.0.9b with a custom RefSeq-based database (41). In order to get longer contigs, all unclassified reads of SIA, MDA, and MTG libraries were mixed together and *de novo* assembled using MEGAHIT version 1.1.3 (59), while those of MTT library were coassembled using Trinity version 2.10.0 (60). Contigs of ≥1 kb were retained for reference-based annotation using BLASTn version 2.7.1 and DIAMOND version 0.9.25 (61) searches with e-value cutoff of 1e−5 against the GenBank and UniProt virus divisions. The BLASTn- and DIAMOND-classified sequences were then defined in the final viromic sequence assemblage if they (i) did not match the false references identified in our refined EVRD-nt and EVRD-aa reference databases, which are viral genome sequence contaminants related to host genomes, laboratory components, nonviral organisms, or artifacts (62); (ii) were shared by the two methods with the same or close taxonomic annotation, and/or (iii) had no significant hits to nonvirus nt and nr databases by BLASTn/x searches (e value ≥ 1e−20 and identity ≤ 50%) (47).

### Evaluation of batch effect.

To evaluate the data heterogeneity caused by batch effect, i.e., sample repreparation of MTG and MTT methods, all clean data and the unclassified ones after Kraken2 assignment were compared using MASH version 2.2 (63). We created sketch files from all data with a sketch size of 10,000 and a K-mer size of 21, excluding singleton K-mers. All-to-all comparisons of the clean and unclassified data were respectively performed using these sketch files to generate the MASH distances, and then Ward’s minimum variance clustering of these libraries was implemented based on the distance matrix.

### Performance assessment.

As to assessing the sensitivity and bias of the four methods in virus detection, reads from each library were aligned against the genomes of the five spiking viruses using bowtie2, and the coverage and depth were calculated using samtools version 1.10. To assess the rectum virome of marmots, any contigs of the five spiking viruses were discarded, the remaining contigs were subjected to species-rank virus grouping, and vOTUs of these contigs were obtained using MMseqs2 clustering with sequence similarity threshold of 0.95 and coverage of target of 0.85 (51, 64). All vOTUs were annotated using the best BLASTn/x hit against viral nt/nr reference database and clustered into different Baltimore classifications depending on their type of genome (https://viralzone.expasy.org). To generate a read count table of vOTUs, the reads of each library were normalized to 6 Gb, except the two SIA libraries at viral concentration of 100 copies/μL that generated only about 2 Gb of data (Data Set S1), the numbers of reads matching to vOTUs were counted using bowtie2 and samtools version 1.10 with vOTUs having only one read being removed. In addition, these vOTUs were further clustered at the approximate genus and family levels. Although there is no consensus on the approach and threshold used in higher taxonomic rank clustering, and classification above the family level requires more signatures than the only sequence similarity matrix (51, 55, 65), Camarillo-Guerrero et al. found that a boundary at the subgenus level of sequences can be achieved at 90% similarity over a 75% aligned fraction (24), and viruses can be roughly clustered to the family rank with an amino acid (aa) similarity threshold of 50% (66). We did not aim to precisely cluster these sequences at higher taxonomic ranks but to assess the stability of relative abundance under different cluster levels and their impacts on the ecological analyses. Accordingly, we clustered nucleotide (nt) and aa sequences using MMseqs2 with 80 and 50% similarity over 70 and 50% coverages for the ranks of genus and family, respectively. The read counts at the genus and family levels were computed by adding these of their vOTU members, with singletons being excluded. vOTUs accumulation curves of the MDA, MTG, SIA, and MTT libraries were assessed using vegan package in the R environment (version 3.6.2) with random method under 10,000 permutations. To examine the beta diversities between MDA, MTG, SIA and MTT libraries, a Bray-Curtis dissimilarity matrix of the vOTUs table was calculated and subjected to principal coordinate analysis (PCoA). vOTUs of different Baltimore group were respectively projected onto the ordination of libraries. Shannon and Simpson diversity indices of each data set for the four methods was deduced using “diversity” function with vOTU table. The CVs of certain indices were calculated by division of standard deviation with mean of the index. To understand the effects of UDS on virus detection and viromic profiling, one library for each of the four techniques at the viral concentration of 10 gene copies/μL was selected to additional sequencing, which eventually generated 47.1, 36.8, 23.3, and 40.7 Gb of data for MDA, MTG, SIA, and MTT, respectively. UDS data were randomly subsampled to 6 Gb and to its serial folds, which were independently replicated three times with exception of the last data size. Subsampled data were subjected to the same analyses as described above.

### Availability of data and materials.

All sequence reads generated in this study are available in the NCBI SRA database under BioProject accession no. PRJNA750309. The essential codes used to generate the viromic data and ecological results were the same as that used in our previous study with a minor adjustment (47). vOTUs in FASTA format are available from the Figshare website at: https://doi.org/10.6084/m9.figshare.16591670.
