# Supplementary material for: Comprehensive Evaluation of RNA and DNA Viromic Methods Based on Species Richness and Abundance Analyses Using Marmot Rectal Samples
Source: mSystems. 2022 Jul 14;7(4):e00430-22. doi: 10.1128/msystems.00430-22 (PMC9426427; doi:10.1128/msystems.00430-22)
Supplement: FIG S1 [file msystems.00430-22-sf001.pdf]

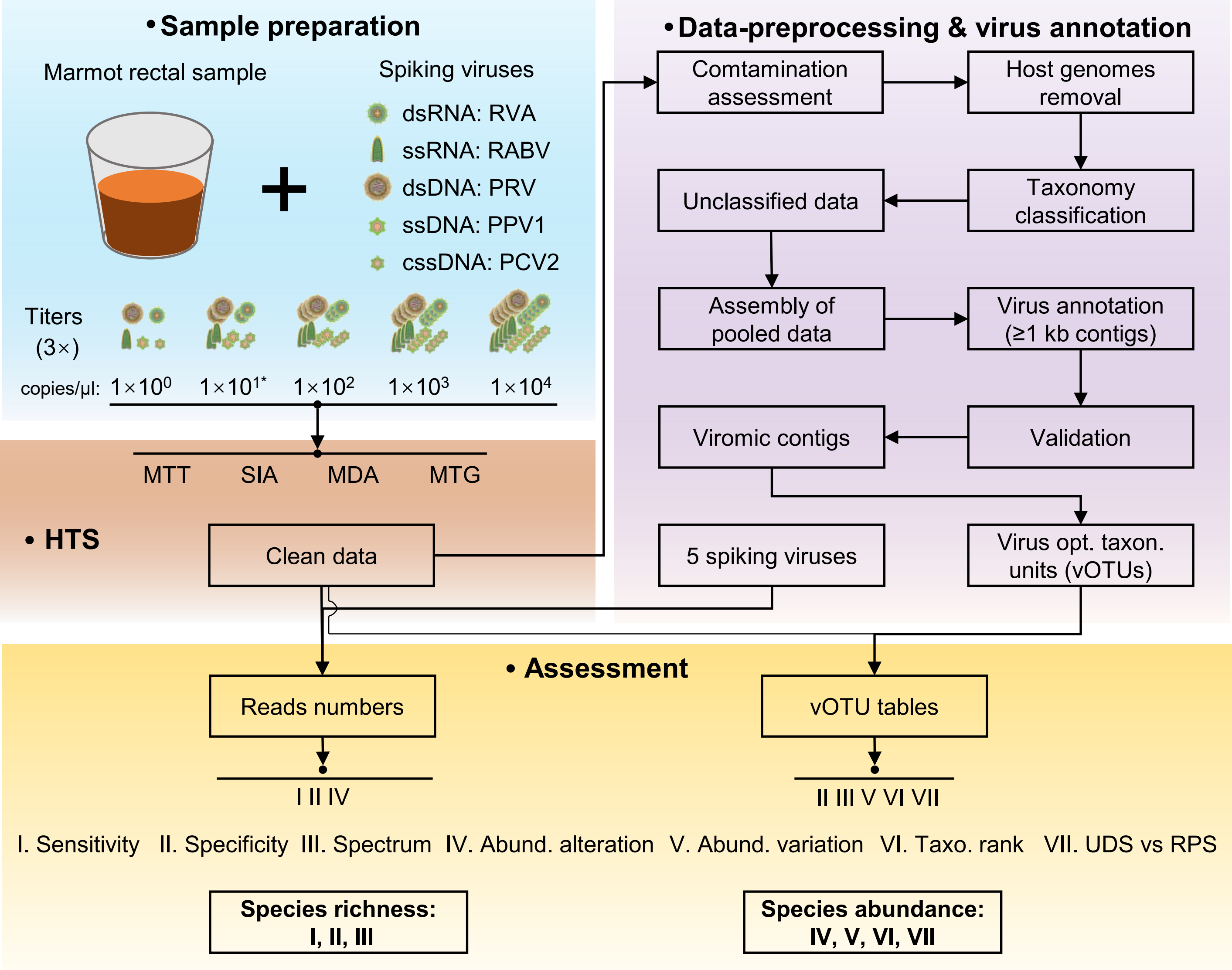
• Sample preparation
Marmot rectal sample
Spiking viruses
dsRNA: RVA
ssRNA: RABV
dsDNA: PRV
ssDNA: PPV1
cssDNA: PCV2
Titers (3×)
copies/μl: 1×10^0 1×10^1* 1×10^2 1×10^3 1×10^4
MTT SIA MDA MTG
• HTS
Clean data
• Data-preprocessing & virus annotation
Comtamination assessment
Host genomes removal
Taxonomy classification
Unclassified data
Assembly of pooled data
Virus annotation (≥1 kb contigs)
Validation
Viromic contigs
5 spiking viruses
Virus opt. taxon. units (vOTUs)
• Assessment
Reads numbers
vOTU tables
I II IV
II III V VI VII
I. Sensitivity II. Specificity III. Spectrum IV. Abund. alteration V. Abund. variation VI. Taxo. rank VII. UDS vs RPS
Species richness: I, II, III
Species abundance: IV, V, VI, VII
