# Supplementary figures and images for: Comprehensive Evaluation of RNA and DNA Viromic Methods Based on Species Richness and Abundance Analyses Using Marmot Rectal Samples
Source: mSystems. 2022 Jul 14;7(4):e00430-22. doi: 10.1128/msystems.00430-22 (PMC9426427; doi:10.1128/msystems.00430-22)

Mash distance of clean data

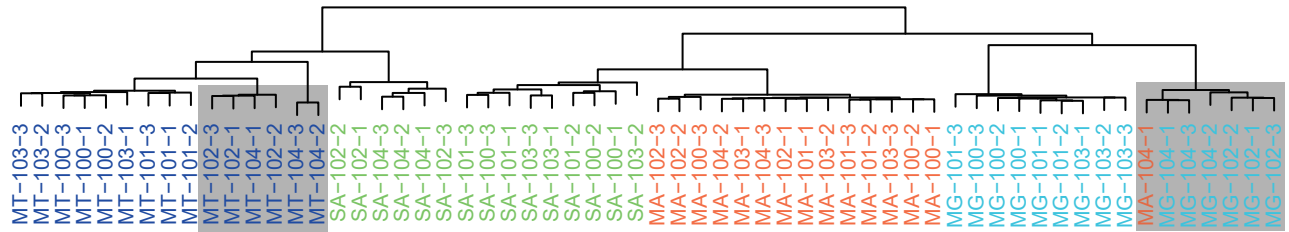

Mash distance of unassigned data

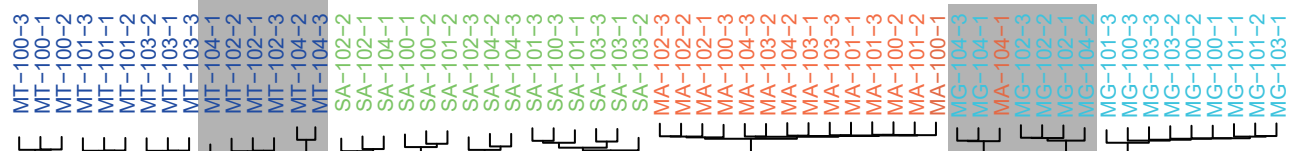

Supplement: FIG S2 [file msystems.00430-22-sf002.pdf]

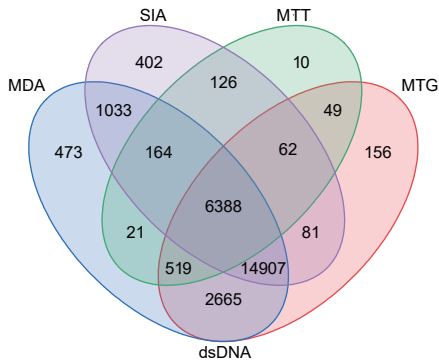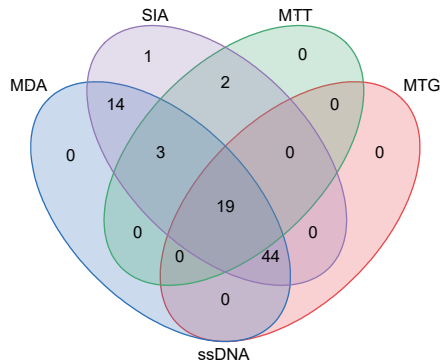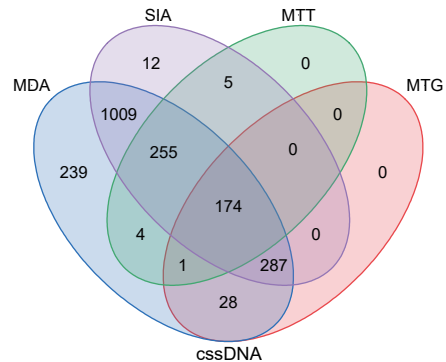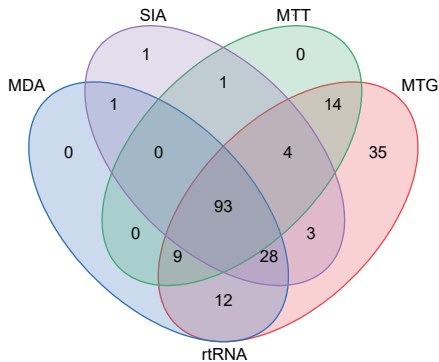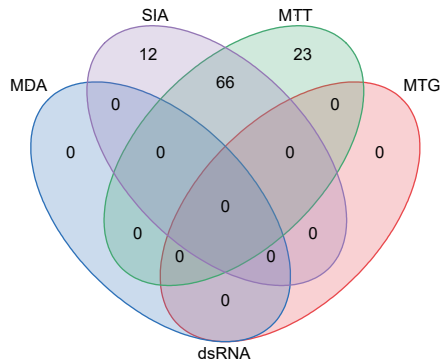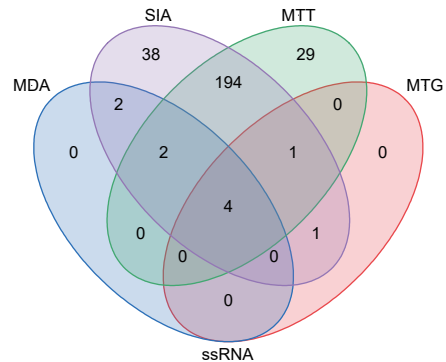

Supplement: FIG S3 [file msystems.00430-22-sf003.pdf]

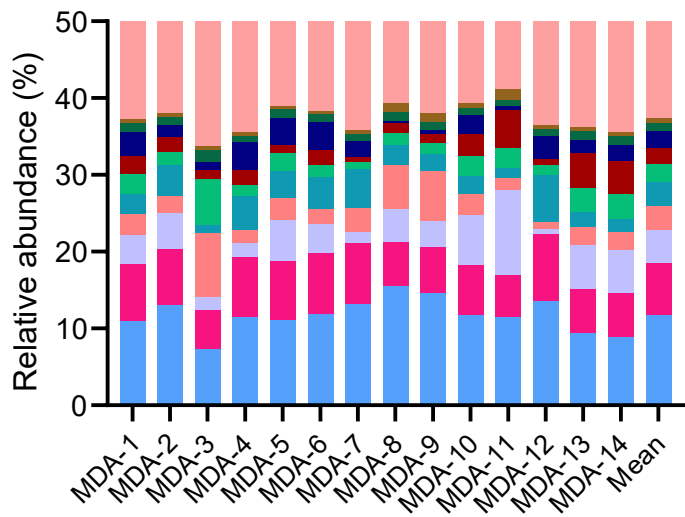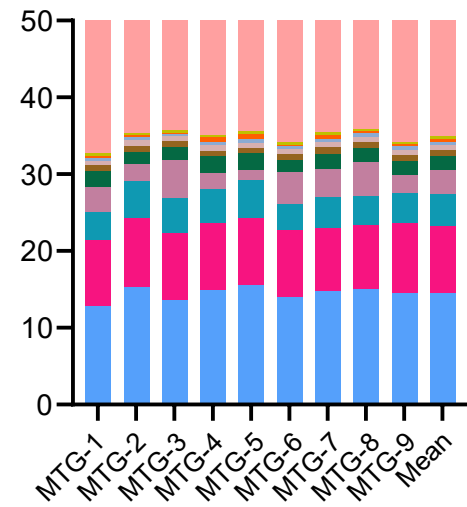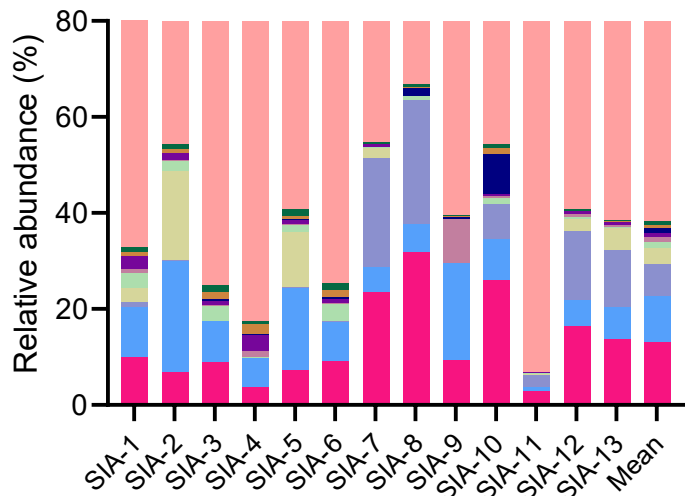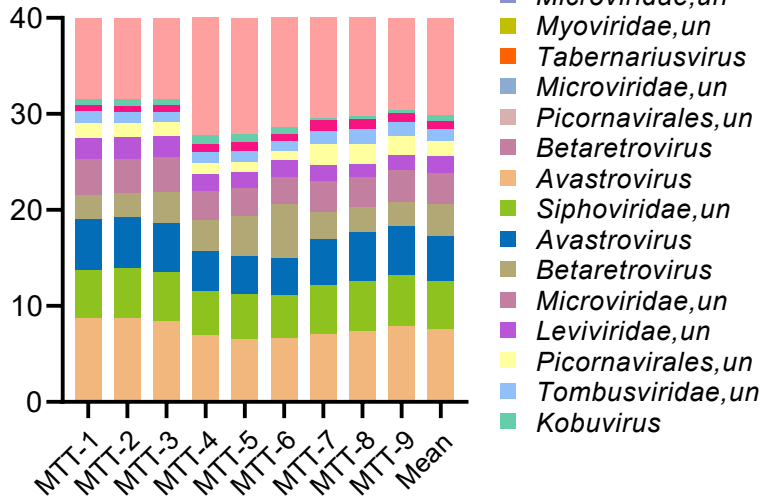

Supplement: FIG S4 [file msystems.00430-22-sf004.pdf]
